# Characteristics of airborne particles emitted from typical indoor combustion sources

**DOI:** 10.3389/fpubh.2025.1540166

**Published:** 2025-02-19

**Authors:** Chen Geng, Xinyuan Wu, Tao Wang, Hongbo Fu

**Affiliations:** ^1^Shanghai Key Laboratory of Atmospheric Particle Pollution and Prevention, Department of Environmental Science and Engineering, Institute of Atmospheric Sciences, Fudan University, Shanghai, China; ^2^Collaborative Innovation Centre of Atmospheric Environment and Equipment Technology (CICAEET), Nanjing University of Information Science and Technology, Nanjing, China; ^3^Institute of Eco–Chongming (SIEC), Shanghai, China

**Keywords:** indoor air, combustion emissions, particulate matter, size distribution, health risk

## Abstract

Combustion is an important source of indoor emissions, and exposure to combustion emissions not only concerns the quality of life of individuals but also directly affects the overall health level of society. To date, very few studies have examined the size-resolved emission characteristics of airborne particulate matter (PM) emitted from indoor sources. The study examined PM emissions from the specified combustion sources. PM concentrations and emission factors for metals and polycyclic aromatic hydrocarbons (PAHs) were analyzed under identical burning durations. Particle size distributions were determined, and dissolved organic matter (DOM) components were characterized using fluorescence spectroscopy. Health risk assessments were conducted to identify major carcinogenic risks among the emitted components. The results revealed distinct trends in PM concentrations and emission factors among the combustion sources, with cigarettes exhibiting the highest levels followed by mosquito coils and candles. The peak diameters of PM number concentration were found to be 68.5 nm for mosquito coils, 105.5 nm for cigarettes, and 201.7 nm for candles. Fine fraction (PM_0.056–3.2_) had significantly higher emission factors than coarse fraction (PM_3.2–18_), with the highest emission factor observed within the particle range of 0.18-0.32 μm. DOM from burning mosquito coils and cigarettes comprised two primary components: a protein-like (C1) and a humus-like (C2) fluorescent component. Health risk assessments indicated that chromium and benzo[a]pyrene posed the greatest carcinogenic risks among metals and PAHs in typical indoor combustion environments. Our results were primarily helpful to determine the characteristics of the PM from combustion emissions and also significant to ensure public health protection, especially for people who usually spend time indoors.

## Graphical abstract

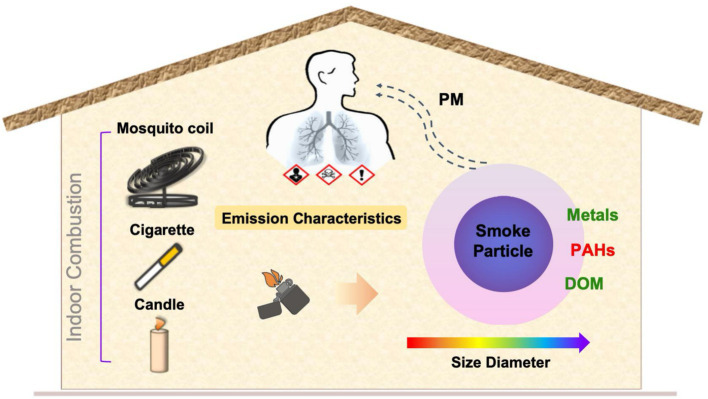

## 1 Introduction

Combustion activities are a major source of airborne particulate matter (PM) indoors (1), and the particles released during combustion are associated with a range of adverse health effects, such as respiratory diseases, cardiovascular problems, asthma, and allergies (2–4). Since people spend more than 80% of their time indoors, exposure to PM from these sources poses significant health risks, as particles can accumulate in indoor air over time. Therefore, it is crucial to conduct a systematic examination of the properties of PM emitted from indoor combustion sources.

The most significant particle sources in indoor environments include tobacco smoking, cooking, kerosene heating, and wood burning (5–7). Among them, it is noteworthy that the majority of households do not have specialized particle removal devices when burning candles, mosquito coils, and cigarettes. In light of the rapid generation of substantial quantities of PM from such combustion, extensive studies of these indoor combustion emissions and health risk assessment have been carried out widely (8, 9). The average concentrations of PM in a 35 m^3^ closed room were estimated to increase up to 1,630 μg·m^−3^, by smoking three cigarettes, and 2,510 μg·m^−3^, by burning 8 cm mosquito-repellent incense (10). Manoukian reported that the emission of PM increased dramatically during the combustion, up to 9.1 × 10^4^ and 22.0 × 10^4^ units·cm^−3^ for incenses and candles, respectively (11). However, majority of the existing studies focused on total concentrations of emitted PM (12), leaving significant gaps on the size distribution scale, which significantly influence their biological effects upon inhalation (13, 14).

Notably, since indoor combustion may emit small particles, which can be high in number but contribute very little to mass, there is a high probability of penetration into the deeper parts of the respiratory tract (15). They also contain high levels of metals and polycyclic aromatic hydrocarbons (PAHs) (16), which has been a subject of increasing concern. As two important chemical components of PM emitted from combustion process, metals and PAHs have been found to be significant factors of health effects, potentially impacting respiratory and nervous systems (17). The substantial quantities of PM emitted from mosquito coil combustion were detected, which include heavy metals such as cadmium (Cd), zinc (Zn), and lead (Pb), alongside PAHs (18), and exposure to the mosquito coil smoke poses both acute and chronic health risks (19). During steady-state burning, candles release a mix of large carbon particles and ultrafine organic particles, with 8–23% of wick lead emitted as fine particles into the air and the rest remaining in wax. Derudi et al. (20) determined the emission factors of PAHs, aromatic species, and PM from container candles comprising different paraffin waxes for burning and emphasized the high carcinogenic risks of PAH levels exceeding the WHO standard. Additionally, cigarette smoke is recognized as a major indoor air problem globally due to its high content of heavy metals and PAHs (21, 22). Goel et al. (16) assessed the highest carcinogenic risk in cigarette, reassuring the health hazard from smoking. The toxicity caused by inhaling PM carrying metals and PAHs is not easily decomposed, and prevention is the key to controlling metal and PAH pollution. Thus, the detection of the composition-emitted characteristics of these PM from indoor combustion has a certain meaning to supplement the data.

Additionally, the dissolved organic matter (DOM) is an important carrier in the conversion process of ions, metals, and other substances. From a microscopic perspective, the molecular structure and functional groups of organic pollutants further influence toxicological effects. But to our knowledge, no study has been performed regarding the structure of DOM within the PM emitted from indoor combustion sources. Furthermore, previous studies have scarcely focused on the chemical composition across size-segregated particles from combustion, and the scope of indicators incorporated in the health assessments is inadequate (23). Consequently, there is still a scarcity of systematic studies that span from the initial concentration characteristics of emissions to the subsequent chemical properties and health effects of collected samples, particularly a lack of comparison under a size distribution scale.

In this study, the sampling room was selected in a typical Chinese university in Shanghai to obtain the characteristics of the PM emitted from mosquito coil, cigarette, and candle combustion, respectively. We determined the physical concentrations, the size-segregated chemical characteristics of the PM, and the carcinogenic risk of heavy metals and PAHs. This study was performed to (i) characterize the size-resolved temporal evolution of PM emitted from the combustion of mosquito coil, cigarette, and candle; (ii) quantify the emission factors of metals, DOM, and PAHs within these size segments; and (iii) compare the distribution characteristics of emissions across diameter sizes and conduct carcinogenic risk assessment.

## 2 Experimental section

### 2.1 Sampling

The monitoring and sampling were conducted in an office at Fudan University (31°18′N, 121°29′E), China, during November 2022. The sampling office dimension was 4.6 m × 4.0 m × 3.2 m, with the combustion apparatus centrally placed inside. Supplementary Figure S1 shows the sampling diagram for indoor combustion experiment. The combustion test system in this study consists of two fans (to ensure uniform air mixing), an airflow unit with humidity and temperature recorder, and a smoke generation (smoldering) unit for burning mosquito coil, cigarette, and candle. Before the start of the experiment, the experimental room was cleaned to eliminate the influence of external factors on the results of the experiment. During the experiment, mosquito coils, cigarettes, and candles were in a free burning state, and cigarettes were released with side-stream smoke. Each combustion process was carried out for 1 h, and the windows and doors were kept closed throughout the test to minimize external interference. In the experiment, mosquito coils, cigarettes, and candles used for combustion were sealed and stored under ambient temperature and light avoidance conditions to ensure the consistency and accuracy of experimental data. The mass of combustion materials was weighed and recorded before and after combustion to calculate the combustion emission factor. Additionally, we used an emission factor to represent the enrichment extent which is defined as the ratio of the mass of metals and PAHs to the consumption mass of combustion materials (24, 25). The size-segregated samples were collected on 47-mm quartz filters (PALLFLEX, USA) using a 10-stage micro-orifice uniform deposit impactor (MOUDI, MSP Corp, USA; Model 110-R) with a flow rate of 30 L·min^−1^ for 1.5 h. And at each sampling site, 10 samples were collected during 11:30 to 13:00 on weekdays. The quartz filters were pre-baked at 500°C for 4 h in a muffle furnace to remove water and organic traces. The cascade impactor divided aerosols into 10 cutoff diameters: 0.056–0.10 μm, 0.10–0.18 μm, 0.18–0.32 μm, 0.32–0.56 μm, 0.56–1.0 μm, 1.0–1.8 μm, 1.8–3.2 μm, 3.2–5.6 μm, 5.6–10 μm, and 10–18 μm.

### 2.2 Size distribution analysis

Measurements of sub-micrometer particle concentration and size distribution ranging from 14.1 to 661.2 nm were conducted using a Scanning Mobility Diameter sizer (SMPS, Model 3,936) manufactured by TSI, Inc. The SMPS comprised an electrostatic classifier (EC 3082, TSI, USA), a differential mobility analyzer (DMA, Model 3,082, TSI, USA), and a condensation particle counter (CPC, Model 3,772, TSI, USA). During the measurement, the DMA sheath sample flow ratio was set to 10:1, and the scan time was set to 300 s. The SMPS system was able to scan the concentration in the range of 1-10^8^·cm^−3^, and the analyzer software inverted the measured data into aerosol diameter size and concentration profiles.

### 2.3 Component analysis

#### 2.3.1 Metals

A quarter of the filters with 4 mL of concentrated HNO_3_ and 1 mL of concentrated HF were taken and digested in a polytetrafluoroethylene high-pressure digestion tank at 180°C to dissolve completely, the liner was removed after cooling, heated at 180°C until the acid completely volatilized, 2% HNO_3_ residue was dissolved and fixed to volume for analyzing the total concentration of the trace elements in the sample. The treated sample solution was transferred to a sample bottle and stored at 4°C for the measurement of total metals. Another quarter of the filters were taken for ultrasonic extraction with 10 mL of DI water for 1 h and then filtered using 0.45 μm filters. Then, 5 mL of the extract was taken and acidified with 2% HNO_3_ content, and the analysis was completed within 48 h (26). In this experiment, 18 metals were detected: Na, Mg, K, Ca, Hg, Mo, Ba, Cr, Pb, As, Mn, Ni, V, Co, Ag, Cd, Sb, and U. The total concentration of the metals in the sample was analyzed using an Agilent 7500c ICP-MS. To ensure the quality of the analysis, a standard solution close to the sample concentration was added for every 10 samples analyzed. Quality assurance and control of the ICP–MS was guaranteed by the analysis of a certified reference standard, NIST SRM-1648 (27). The resulting recoveries fell within ±10% of the certified values for majority of the elements, except for Na, As, and Sb (±15%). All samples were analyzed in duplicate for quality assurance/quality control of laboratory analyses.

#### 2.3.2 Polycyclic aromatic hydrocarbons

Half of the filters were taken for ultrasonic extraction with 3 mL of methanol in an ultrasonic bath twice for 30 min each time, and ice was added to maintain the extraction temperature under 25°C. Then, the combined extracts were filtered using 0.22 μm filters and evaporated under a gentle stream of nitrogen (N_2_, purity ≥99.99%) until a measure of 0.2 mL was obtained. The concentrated samples were stored at −20°C for further analysis, and the detection was completed within 48 h (18). The concentrated samples were analyzed using an Agilent 7890B gas chromatographer coupled to an Agilent 7000D mass spectrometer with an electron impact (EI) ion source. The column was HP-5MS (30 m × 0.25 mm × 0.25 μm) provided by Agilent. The column temperature program was initiated at 80°C, increased to 170°C at 20°C/min (held for 6 min), and then increased to 300°C at 5°C/min (held for 2 min). A capillary column was used for separating PAHs. Helium (He, 99.999% purity) was used as a carrier gas at a flow rate of 1 mL/min. The mass spectrometry analysis adopted the selective particle detection (SIM) scanning mode, with a solvent delay time of 5 min and an ion source temperature of 280°C. This study experiment detected 16 PAHs: naphthalene (Nap), acenaphthene (Acy), acenaphthylene (Ace), fluorene (Flo), phenanthrene (Phe), anthracene (Ant), fluoranthene (Flu), pyrene (Pyr), benzo[a]anthracene (BaA), chrysene (Chr), benzo[b]fluoranthene (BbF), benzo[k]fluoranthene (BkF), benzo[a]pyrene (BaP), dibenzo[a,h]anthracene (DahA), indeno[1,2,3-cd]pyrene(IcdP), and benzo[g,h,i]perylene(BghiP). The overall analytical procedure was previously validated by systematic recovery experiments using the standard reference material. All samples were analyzed in duplicate for quality assurance/quality control of laboratory analyses. PAH QA/QC was performed by field and laboratory blanks and standard spiked recoveries. PAHs were identified relative to internal standards. Recovery of PAHs and internal standards varied from 78% (Chr) to 131% (BbF).

#### 2.3.3 Dissolved organic matter

Half of the filters were taken for ultrasonic extraction with 5 mL of methanol in an ultrasonic bath for 60 min, and ice was add to maintain the extraction temperature under 25°C. The samples were filtered using 0.45 μm filters, and the detection was completed within 24 h. Three-dimensional excitation-emission matrix spectra (3DEEMs) were measured using a fluorescence spectrophotometer system (Aqualog, manufactured by HORIBA, Japan), with an ozone-free xenon arc lamp of 150 W serving as the excitation light source. The UV–visible absorption spectrum was measured using a 10 mm quartz cuvette, with a scanning wavelength range (excitation wavelength: Ex) of 200–800 nm and an integration time of 0.1 s. After obtaining the fluorescence spectrum of the DOM, the instrument automatically deduced the spectrum of a blank sample from the 3DEEM data of the samples to eliminate Raman scattering. Additionally, Rayleigh scattering was removed using the drEEM software package within MATLAB to ensure quality (28). All samples were analyzed three times for quality assurance/quality control of laboratory analyses.

### 2.4 Carcinogenic risk assessments

Given the potential risk of lung cancer associated with the exposure to heavy metals (29) and the adverse effects on the respiratory system posed by PAHs (30), a thorough evaluation of the two contaminants was conducted as part of the carcinogenic risk assessment. The carcinogenic risk for a receptor exposed via inhalation pathway could be calculated by the method provided by the US Environmental Protection Agency according to Equation 1 (31).


(1) CR=IUR×EC

where CR is the carcinogenic risk; IUR is the inhalation unit risk (μg·m^−3^)^−1^, provided by the USEPA; and EC is the exposure concentration (μg·m^−3^), calculated using the Equation 2:


(2) $$EC=\left(CA\times ET\times EF\times ED\right)/AT$$

where CA is the contaminant concentration in air (μg·m^−3^); ET is the exposure time (hours·day^−1^); EF is the exposure frequency (days·year^−1^); ED is the exposure duration (years); and AT is the averaging time for exposure (days).

Based on the definitions and classifications of compound toxicity by the International Agency for Research on Cancer (IARC), Cr, Ni, Pb, Cd, and As were identified as carcinogenic compounds. They were also reported as carcinogenic elements in cigarette, candle, and mosquito coil smoke (32). According to previous studies (33, 34) and the data we detected, heavy metals, c-PAHs (BaA, Chr, BbF, and BaP), and other relative parameters used are shown in Supplementary Table S1.

The carcinogenic risks lower than 10^−6^ are considered negligible, and risks above 10^−4^ are not accepted by majority of the international regulatory agencies (35, 36). An incremental lifetime cancer risk (ILCR) value below 10^−6^ signifies a negligible risk of cancer, while a range of 10^−5^ to 10^−4^ indicates the presence of a moderate carcinogenic risk. Conversely, an ILCR exceeding 10^−4^ denotes a considerably high carcinogenic risk.

### 2.5 Statistical analysis

Statistical analysis was performed using Origin 2021 software (OriginLab Corp., USA). All correlation analyses were performed using SPSS 24.0 software (IBM Corp., USA), with a significance level of 0.05. Tukey’s honestly significant difference (HSD) test (*p* = 0.05) was employed to assess the significance of differences among each component. In addition, we used the coefficient of divergence (COD) to analyze the difference in the chemical compositions of the combustion sources.

## 3 Results and discussion

### 3.1 PM number and mass distribution

**Figure 1:**
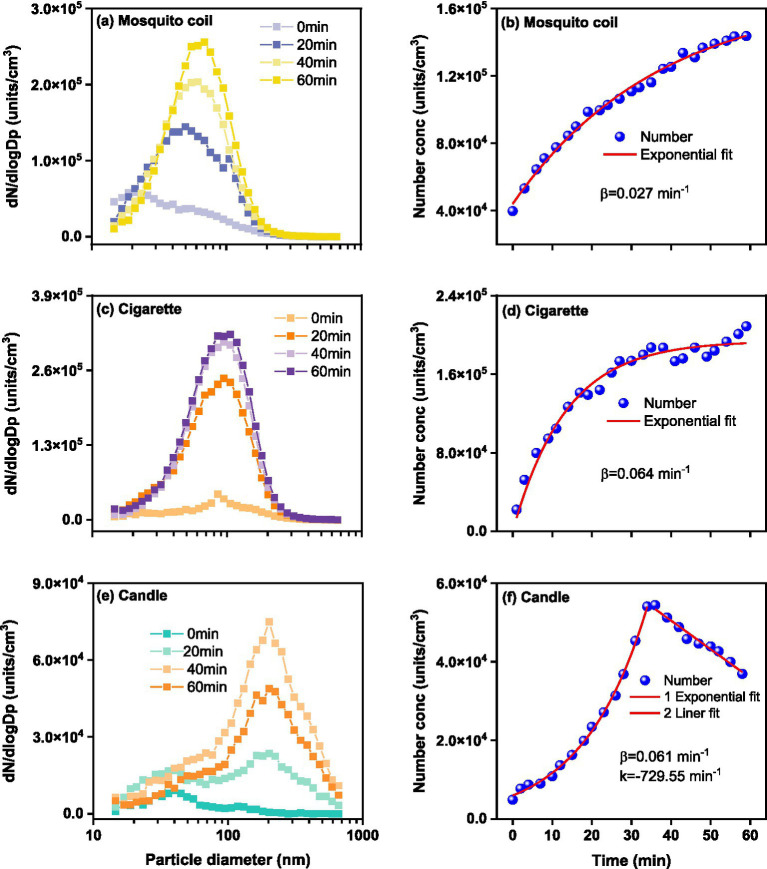
Size-resolved PM number concentration during **(A)** mosquito coil, **(C)** cigarette, and **(E)** candle combustion, and fitted lines for **(B)** mosquito coil, **(D)** cigarette, and **(F)** candle combustion as a function of measurement time.

Figure 1 shows the number concentrations of size-resolved PM throughout the combustion. The increase in PM concentrations exhibits distinct patterns. The total number concentrations of PM emitted from mosquito coils and cigarettes gradually increased during the combustion process. For the mosquito coil burning, high PM concentration (1.44 × 10^5^ units·cm^−3^) was observed with 80% of the total PM in the 10–200 nm size range, which was in proximity to 1.30 × 10^5^ (37). The PM emitted from cigarette combustion also showed relatively high total number concentrations (2.09 × 10^5^ units·cm^−3^), with about 80% of the total within the size range of 10–300 nm. For the candle-derived PM, high concentrations (5.45 × 10^4^ units·cm^−3^) were observed, with uniform distribution within the size range of 10–600 nm, which was slightly lower than 6.90 × 10^4^ units·cm^−3^ (38). The PM number concentration can be fitted to a first-order exponential equation and α is the increase exponent (min^−1^). The number increase exponent values are 0.064 min^−1^ and 0.061 min^−1^ for the PM emitted from cigarette and candle burning, respectively, with both being much higher than that of mosquito coil burning, 0.027 min^−1^. Specifically, the PM concentration for mosquito coil and cigarette burning increases progressively over time for majority of the diameter sizes. For cigarette combustion, there is a significant initial surge within the first 20 min, followed by a gradual increase. Meanwhile, compared to the subsequent 20–40 min interval, the PM generated by mosquito coil burning slightly increased in the first 20 min. Conversely, for candle burning, the PM concentration sharply increased in the first 35 min, reached its peak at approximately 35 min, and subsequently exhibited a decline. The attenuation of PM observed during candle burning is attributed to the further combustion of melted wax, albeit with most PM being emitted during the initial burning stages. Among the three sources, the peak diameter of the number concentration of PM produced by mosquito coil combustion is 68.5 nm, while those of cigarette and candle combustion were 105.5 nm and 201.7 nm at 60 min, respectively. In addition, we observed that with the increase of the particle number due to accumulation, the peak diameter tended to gradually increase with time except for candle combustion. For example, regarding mosquito coil combustion, the peak diameter of number concentration was 49.4 nm after 20 min, 61.5 nm after 40 min, and 68.5 nm after 60 min. For cigarette combustion, the peak diameter of number concentration was 94.7 nm at 20 min and 40 min, respectively, and 105.5 nm at 60 min. This phenomenon indicates that at a shorter suspension time, the fine nanosized particles or fine particles may collide with each other and generate larger particles. In general, the type of combustion source largely affects the level and variation of indoor PM.

As shown in Supplementary Figure S2, the total mass concentration of ultrafine PM emitted from cigarette combustion exhibits an exponential increasing trend over time, characterized by an α value of 0.038, peaking at 184.57 μg·m^−3^. The mass concentration of PM emitted from candle burning increases with diameter, peaking at 514.7 nm. However, after 300 nm, there is still a significant amount of mass, corresponding to the minimum amount of PM, indicating that PM >300 nm contributes mainly to the PM mass emitted from candle combustion. The increasing trend of the mass concentration of PM emitted from candle burning also exhibits an exponential trend to ~35 min, with an α value of 0.079 min^−1^, peaking at 615.98 μg·m^−3^. Moreover, the total mass concentration of PM emitted from mosquito coil burning exhibits a linear increasing trend within time, with a linear increase rate (k) of 0.68 min^−1^, peaking at 56.16 μg·m^−3^, which is notably lower than the average PM_1_ concentration of 214.0 μg·m^−3^ (39). The trend in temporal variation of PM emissions from mosquito coil burning diverges between mass and number results, likely influenced by the differing condensation and deposition processes of nanoparticles within indoor environments. It can be noted that the PM emitted from candle burning showed the lowest number concentration for all the combustions considered, but the highest mass concentration, which can be reasonably ascribed to the mass concentration limitation and larger nanoparticle diameter. It can be observed that PM emitted from cigarette burning dominates the mass concentration within the diameter range of 14.6–310.6 nm, with the peak diameter remaining relatively constant at ~145.9 nm, consistent with a past cigarette combustion experiment (40), which reported a peak particle size of 150.9 nm in combustion process. Similar to the number concentration, the mass concentration of ultrafine PM emitted from mosquito coil combustion is also predominantly concentrated in the range of 14.6–201.7 nm, with a stable peak size of 117.6 nm. These findings demonstrate that both size distribution and emission concentrations of PM are dependent on the indoor combustion source.

### 3.2 Metal emission characteristics

#### 3.2.1 Emission factors of metals

**Figure 2:**
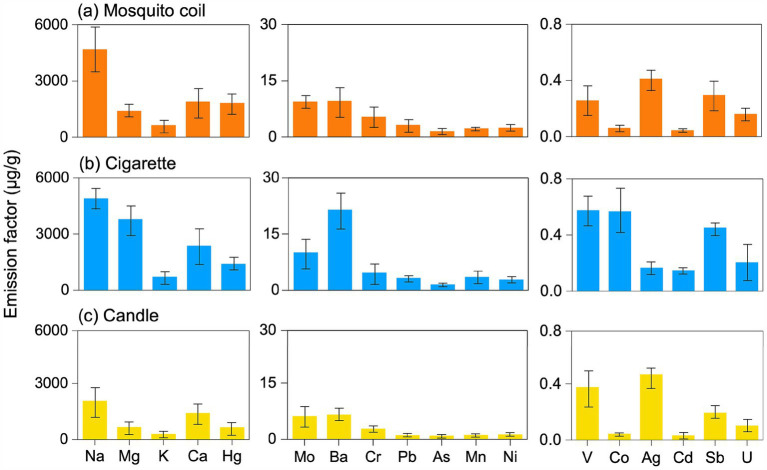
Emission factor of the metals in the PM emitted from **(A)** mosquito coil, **(B)** cigarette, and **(C)** candle combustion.

As shown in Figure 2, the emission factors of Na, Mg, K, Ca, and Hg were the highest among the three combustion sources, followed by Mo, Ba, Cr, Pb, As, Mn, Ni, and then V, Co, Ag, Cd, Sb, and U. The results of the sum emission factor are shown in Supplementary Table S2. Significant differences were observed among the chemical compositions of the three combustion sources (*p* < 0.05). K, being the hallmark element of biomass combustion, is prominently present on the PM emitted from mosquito coils, cigarette, and candle burning, underscoring the consistency across different combustion sources. The total emission factors of metals collected from three combustion sources indoor revealed the following trend: cigarette > mosquito coil > candle, except for Hg, Cr, and Ag. The emission factors of Hg, Cr, and Ag from mosquito coil burning were 477.57, 0.73, and 0.25 μg·g^−1^ higher than those from cigarette burning, respectively. We speculate that specific ingredients used for mosquito repellency contain Hg, and they volatilize and accumulate onto PM, which reflected as the highest emission factor of 1819.05 μg·g^−1^. Consistent with prior cigarette burning study in real indoor spaces (41), the metals most frequently associated with PM from cigarette burning were Na, Mg, and Ca as well as heavy metals such as Cr, Pb, Mn, Ni, and Co. In addition, smoking was found to slightly increase the enrichment of K and As in majority of the PM segments, as well as the enrichment of V, Co, and Ni in the coarse fraction (42). Tobacco smoke in an office increased 11–24 and 8.4–22 times the total concentrations of five carcinogenic elements (Cr, Ni, As, Cd and Pb) in PM_10_ and PM_2.5_, respectively (43). It has been documented that the average Cr, Ni, As, Cd, and Pb levels in cigarette materials are 1.43, 1.26, 0.09, 0.65, and 0.27 μg·g^−1^, respectively (44). The relatively insignificant emission factors of metal elements, notably Na, Mg, and Ca, emitted from candles are most probably attributable to the inherent purity of paraffin wax, the essential constituent of candles, which is devoid of abundant metallic impurities (45). The combustion of mosquito coils could generate PM containing heavy metals such as Cd, Pb, and Hg (46). The concentrations of certain metals were higher in specific sources. For instance, plant ashes, a common mosquito coil ingredient, release high levels of Na and Ca during burning. Conversely, candle combustion contributed the least to the enrichment of metals in indoor air due to their raw material being paraffin wax.

#### 3.2.2 Distribution of metals

**Figure 3:**
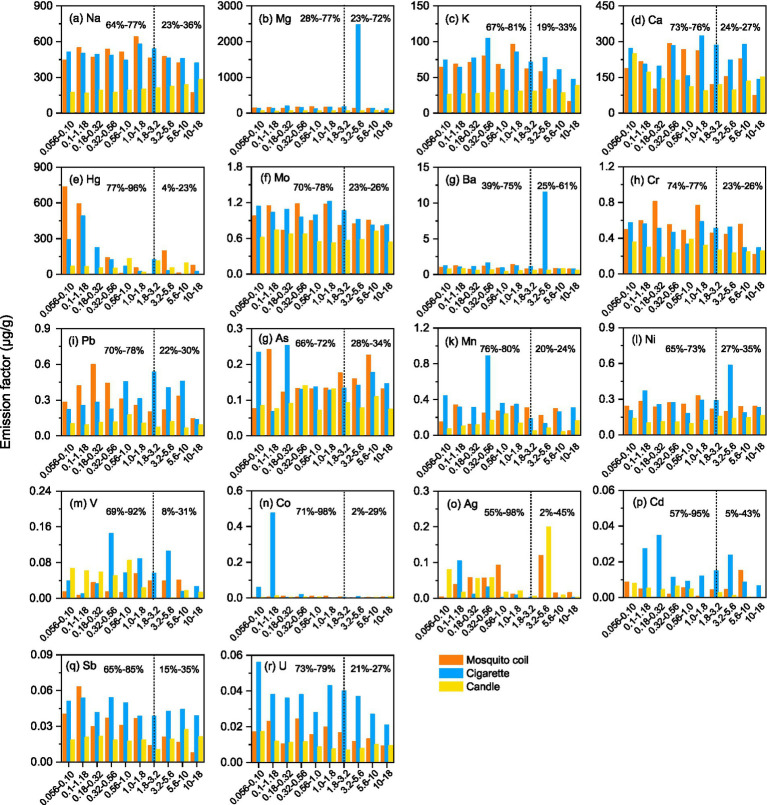
Size distribution of the total metals for three combustion sources; vertical dotted line is the aerodynamic diameter 0.056 -18 μm, the left hand side of the line is the proportion of fine particles, and the right hand side of the line is the proportion of coarse particles. **(a–r)** represent Na, Mg, K, Ca, Hg, Mo, Ba, Cr, Pb, As, Mn, Ni, V, Co, Ag, Cd, Sb, and U,respectively..

Figure 3 shows the size-segregated distribution of metals. It is noteworthy that the size distribution of metals did not exhibit a discernible regularity. The metal in the sampled PM of mosquito coil burning predominantly clustered within the range of approximately 5.6–18 μm. Conversely, for cigarette samples, most metals exhibited densest concentration within the 0.18–0.32 μm range. As for candle burning, a uniform distribution can be observed across each size segment. This scenario further demonstrates that different combustion types have different emissions, and the differences among the three combustion sources from different types were greater than those observed among the different combustion sources in the same type. However, the metal ratios ranged similarly between 5 and 20% for crustal elements such as Mg and Ca, indicating that they are almost equally distributed in each size segment. The metals Mg, Ba, and Ni in cigarettes were significantly enriched at 3.2–5.6 μm, and the concentration of Co suddenly increased at 0.1–1.18 μm, but there is no other evidence that the accumulation rules of Mg, Ba, and Ni are different. The uniform distribution of majority of the metals contrasts with the results of a previous study, which reported that Pb, V, Cr, Co, Mn, Ni, Cu, Zn, As, and Ba increased with decreasing diameter (47).

Given that the patterns of the PM with different sizes will deposit in different areas of the respiratory tract, the accumulation of majority of the coarse particles is easily blocked by the nasal passages and deposited on the head, while fine particles are more likely to deposit in the bronchial area and lungs and contact the blood. Moreover, PM in the bronchial area and lungs is more likely to be a health hazard than that on the head. This suggests that size-segregated dependence of metal enrichment could have significant ramifications for the health impacts of dust aerosols. In this study, we chose stage 7 (1.8–3.2 μm) as the demarcation line of fine fraction and coarse fraction for the determined factors. As shown in Figure 3, among majority of the metals, the proportion of the fine fraction of Co and Ag from cigarette samples was the highest, and it was 94% higher than the coarse fraction. While the proportion of the fine fraction of Sb from mosquito coil burning was the highest, and was 70% higher than coarse fraction, Cd was 90% higher than the coarse fraction for candle burning. Except for Mg (28%) and Ba (39%) collected from cigarette burning, the concentrations of other metals were more highly enriched in fine fraction particles than the coarse fraction particles, which was consistent with the previous studies (47, 48). Harmful components prefer to gather in the fractions that are easier to inhale, thus causing major impacts on human health. In conclusion, despite the absence of a notable size-segregated distribution pattern, metals in PM determined in fine fractions could potentially have a greater impact on public health than those determined in coarse fractions.

### 3.3 PAH emission characteristics

#### 3.3.1 Emission factors of PAHs

**Figure 4:**
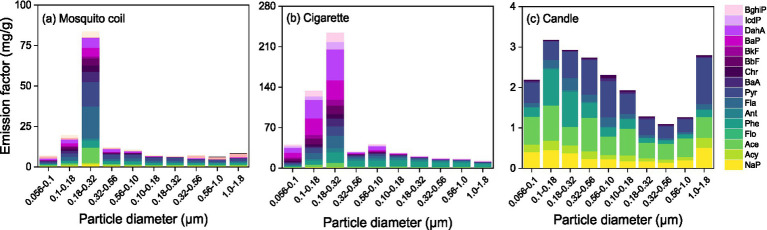
Emission factor of 16 PAHs and size-segregated distribution of PAHs for the PM emitted from **(A)** mosquito coil, **(B)** cigarette, and **(C)** candle combustion.

Figure 4 shows the size-segregated and total emission factors of 16 PAHs. The emission factors of all PAHs from the three combustion sources exhibit the following order: cigarette > mosquito coil > candle, basically consistent with the trend of total metals. Based on the analysis, it is evident that Pyr and Phe constitute a substantially greater proportion than other PAHs. Consequently, this can be designated as a characteristic PAH marker for these combustion sources. Considering the average of the emission factors of most diameters, Pyr, Acy, and Phe are the three major abundant components in candle samples, with emission factors of 6.02 mg·g^−1^, 5.71 mg·g^−1^, and 3.32 mg·g^−1^, respectively, accounting for 68% of total PAHs. As for mosquito coil samples, Pyr, Fla., and Phe are the major abundant components, with emission factors of 31.31 mg·g^−1^, 30.29 mg·g^−1^, and 15.97 mg·g^−1^, respectively, accounting for 46% of total PAHs. Meanwhile, DahA, Phe, and BaP are the major abundant components in cigarette samples, with emission factors of 104.77 mg·g^−1^, 88.61 mg·g^−1^, and 74.64 mg·g^−1^, respectively, accounting for 47% of total PAHs; this is well consistent with the previous study (49). Flo was below the detection limit; therefore a detailed study of Flo was not performed. The concentrations of carcinogenic PAHs (c-PAHs) were 30.55 mg·g^−1^, 142.77 mg·g^−1^, and 28.43 mg·g^−1^ for mosquito coil, cigarette, and candle combustion, respectively. It is noteworthy that our analysis of the candle samples revealed an absence of benzo-compounds and IcdP, which are known to carry a significant carcinogenic risk.

The emission factors of PAHs fall within the range of the calculated values of different indoor combustion sources reported by previous researchers (50), ranging from 0.026 mg·g^−1^to 18 μg·g^−1^, but were higher than 0.06 mg·g^−1^ for wood emission (51). The emission factor of the candle aligns closely with the values reported by Orecchio (49) (2.3–50 mg·g^−1^), and the emission factor of mosquito coil combustion is also similar to the value of 13 mg·g^−1^ reported by Yang et al. (52). The propensity of the oil-containing waxes of candle to produce low levels of PAH emissions can be reasonably ascribed to the oil percentage.

#### 3.3.2 Distribution of PAHs

As depicted in Figure 4, the highest concentration of PAHs is observed within the range of 0.18–0.32 μm, indicating that PAHs are predominantly present in the fine fraction, whereas this trend is less evident in candle burning emissions. Obviously, the distribution of PAHs in mosquito coil combustion exhibits a peak concentration within the diameter size ranges of 0.18–0.32 μm, 0.10–0.18 μm, and 0.32–0.56 μm, with respective mass percentages of 50, 12, and 7%, respectively. As for cigarette combustion, the peak diameter size ranges for PAHs are 0.18–0.32 μm, 0.10–0.18 μm, and 0.056–0.10 μm, each contributing approximately 41, 23, and 7% of the total PAHs, respectively. The peak diameter size ranges for PAHs in candle samples are 0.10–0.18 μm, 0.18–0.32 μm, and 0.32–0.56 μm, each contributing approximately 15, 14, and 13%, respectively. Furthermore, the size distribution of PAHs was consistent with trace metals, which indicates that the concentration of the fine fraction (~70%) was higher than that of the coarse fraction (~30%). This indicates that PAHs have a propensity to accumulate in fine fractions, which facilitates their inhalation by humans and subsequently may pose a series of adverse health impacts. In conclusion, the PAH size distributions in mosquito coil, cigarette, and candle combustion sources exhibit a tendency of highly toxic PAHs accumulating in finer than in coarser particles, posing potential health risks through inhalation.

#### 3.3.3 Characteristic ratios of PAHs

**Figure 5:**
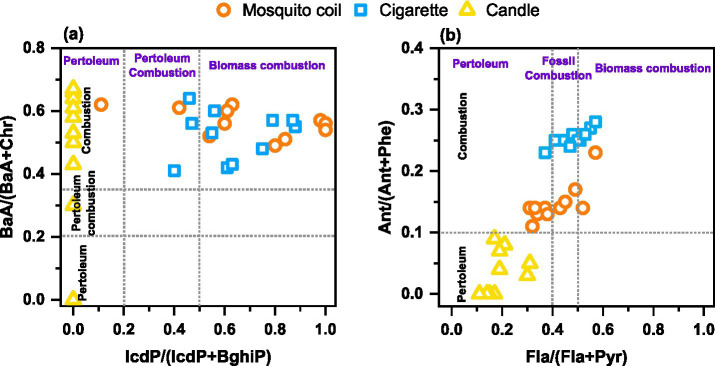
Characteristic ratios of **(A)** BaA/(BaA + Chr) and IcdP/(IcdP + BghiP) and **(B)** Ant/(Ant + Phe) and Fla./(Fla + Pyr) of PAHs in the PM from mosquito coil, cigarette, and candle combustion.

The varying raw materials used in the production of mosquito coils, cigarettes, and candles result in distinct characteristics of PAHs emitted during their combustion. To facilitate further source apportionment, this study investigates the characteristic ratios of PAHs emitted from their combustion. Scholars domestically and internationally widely utilized the ratios of Ant/(Ant + Phe), Fla./(Fla + Pyr), BaA/(BaA + Chr), and InP/(IcdP + BghiP) as a means to determine the sources of PAHs and to identify the various combustion sources (53, 54). The emission characteristic ratios of PAHs derived from various indoor combustion sources are comprehensively presented in Figure 5.

Among the tested mosquito coil and cigarette samples, the Ant/(Ant + Phe) ratios being greater than 0.1 in PM indicate that there is a dominance of combustion in this study. The primary component of candles is industrial-grade paraffin wax, a premium alkane mixture extracted from the waxy fractions of petroleum through processes such as cold pressing or solvent dewaxing. Logically, the burning samples of these candles show a notably lower ratio of Ant/(Ant+Phe) and Fla./(Fla + Pyr), which serves as a clear indicator pointing to a petroleum source. In the context of utilizing characteristic ratios for identification, the IcdP/(IcdP + BghiP) and BaA/(BaA + Chr) values of mosquito coil and cigarette combustion exhibit no discernible differences. Nevertheless, a clear distinction between these two sources can be achieved by employing a threshold of 0.2 for the Ant/(Ant + Phe) ratio, with values equal to or exceeding this threshold indicative of mosquito coil origins and values below that suggesting cigarette origins. This study provides characteristic ratios of PAHs emitted from different indoor combustion sources, particularly those that can accurately distinguish between mosquito coil and cigarette sources. It further refines and optimizes the methodology for source apportionment using PAH characteristic ratios, enhancing the accuracy and reliability of identifying different indoor pollution sources.

### 3.4 DOM characteristics

#### 3.4.1 DOM components

**Figure 6:**
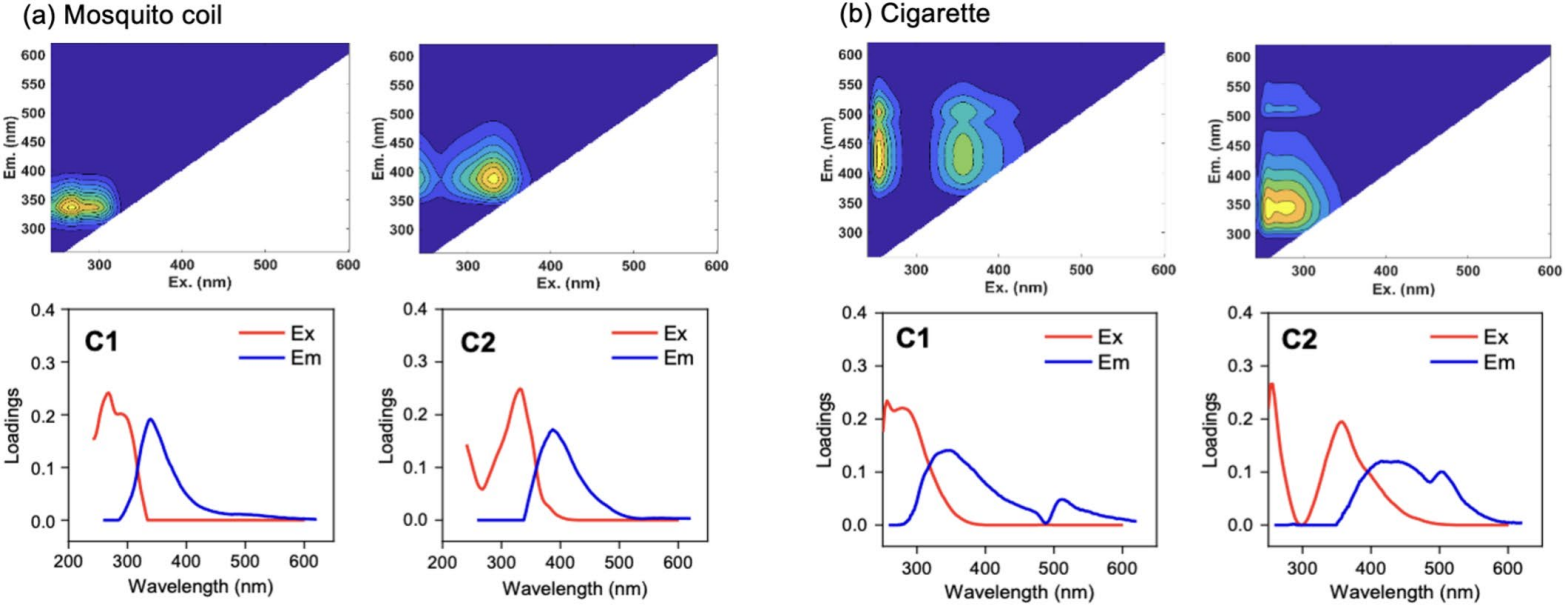
Fluorescent components of PM emitted from **(A)** mosquito coil and **(B)** cigarette combustion.

Based on the obtained consistency in test results, Figure 6 depicts the fluorescence components of DOM in PM emitted from the burning of mosquito coil and cigarette, respectively, and two distinct and effective components were identified in the PM from both sources. For mosquito coil burning, the fluorescence components are Component 1, C1 (Ex/Em = 265/340 nm), and Component 2, C2 (Ex/Em = 330/390 nm) (55). Similarly, in the PM from cigarette burning, the DOM fluorescence components are also C1 (Ex/Em = 250/345 nm) and C2 (Ex/Em = 350/420 nm). These components can be broadly classified into two categories: C1 in both mosquito coils and cigarettes belongs to the protein-like fluorescence component, specifically the tryptophan-like component (Ex/Em = 270–290/320–350 nm) (56), which is primarily free or bound within proteins, suggesting a strong association between microorganisms and such fluorescent substances in the PM emitted from mosquito coil and cigarette burning. C2, on the other hand, belongs to the humic-like fluorescence component (Ex/Em = 300–350/380–420 nm) (57), a commonly encountered DOM component in nature and a typical terrestrial organic matter. Notably, the UV absorbance of DOM and the photo-dependence of organic components in cigarette burning were higher than those in mosquito coil burning. The study revealed that the PM emitted from candle combustion was devoid of DOM, whereas the PM emitted from mosquito coil and cigarette combustion exhibited two unique organic fluorescence components, with higher UV absorbance and photo-dependence observed in cigarette combustion.

Notably, no DOM was detected in the PM emitted from candle burning, which could be attributed to the exceptionally high temperature of the candle flame, consequently increasing the indoor temperature to 30°C during combustion. This heightened environment potentially promotes the volatilization of organic compounds from particle surfaces, dispersing them into the air or fostering their adhesion to indoor surfaces (58, 59). Alternatively, the absence of DOM could stem from the inherent lack of soluble organic matter in the candle itself (60).

#### 3.4.2 Size distribution of DOM

**Figure 7:**
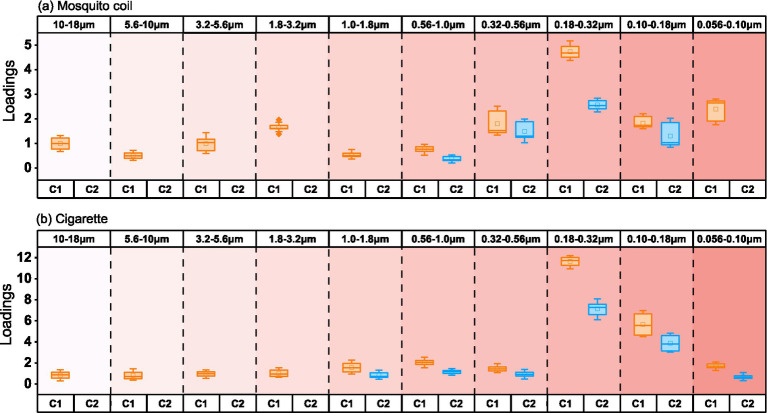
Loading of intensity in size-segregated fluorescent components in PM emitted from **(A)** mosquito coil and **(B)** cigarette burning.

Figure 7 depicts the fluorescence intensity of DOM components (C1 and C2) emitted from the burning of mosquito coils and cigarettes, with (a) and (b) representing mosquito coil and cigarette combustion, respectively, with numbers 1–10 representing the previously mentioned diameter size segments. Analyzing from the perspective of different diameter sizes, within mosquito coil burning, the size distribution of C1 components exhibits a pronounced peak within the range of 0.056–0.56 μm, peaking at 0.18–0.32 μm with a corresponding fluorescence intensity of 4.73, while it is less pronounced in other size ranges. It is evident that C2 components are exclusively present within the specific size ranges of the PM from mosquito coil and cigarette combustion, which are 0.056–1.0 μm and 0.056–1.8 μm, respectively, accounting for 35% of the total fluorescence intensity in both cases. The C2 components exhibit significant fluorescence intensity for DOM components at 0.10–0.56 μm, also peaking at 0.18–0.32 μm with a maximum of 2.57. For PM emitted from cigarette burning, the fluorescence intensity of C1 components is higher at 0.18–0.32 μm and 0.10–0.18 μm, with values of 11.65 and 5.65, respectively, and is less than 2 in other size ranges. Similarly to C1, the fluorescence intensity of C2 components peaks at 0.18–0.32 μm and 0.10–0.18 μm, reaching 7.13 and 3.87, respectively. It can be concluded that DOM is more prone to enrichment in the range of 0.18–0.32 μm of PM.

### 3.5 Carcinogenic risk assessments

**Figure 8:**
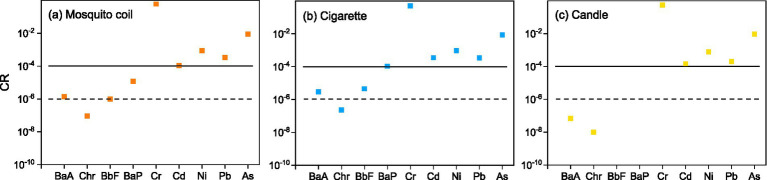
Lifetime carcinogenic risk of the metals (Cr, Cd, Ni, Pb, As) and c-PAHs (BaA, Chr, BbF, and BaP) of **(A)** mosquito coil, **(B)** cigarette, and **(C)** candle combustion.

Figure 8 shows the carcinogenic risk of heavy metals and c-PAHs from three combustion types. In this study, the values of majority of the heavy metals and PAHs from different combustion types revealed the following trend: cigarette > mosquito coil > candle. The integrated risk values of five metals were 6.14 × 10^−1^, 5.18 × 10^−1^, and 5.69 × 10^−1^ corresponding to mosquito coil, cigarette, and candle combustion. The three combustion types were above the tolerance limit, and Cr was found to be the major contributing metal (6.04 × 10^−1^, 5.10 × 10^−1^ and 5.58 × 10^−1^); The values of Ni, Pb, Cd, and As determined from mosquito coil combustion were 1.09 × 10^−4^, 8.68 × 10^−4^, 3.42× 10^−4^, and 8.78 × 10^−3^, while those from cigarette combustion were 3.47 × 10^−4^, 9.64 × 10^−4^, 3.41 × 10^−4^ and 8.64 × 10^−3^ and those from candle combustion were 1.40 × 10^−4^, 7.84 × 10^−4^, 1.99 × 10^−4^ and 9.58 × 10^−3^, respectively, which were all above the tolerance level (1.0 × 10^−4^). The integrated risk value of the four c-PAHs was 1.46× 10^−5^ for mosquito coil combustion, above the acceptable but within the tolerance level; the value was 1.14 × 10^−4^ for cigarette combustion, slightly higher than the tolerance level (1.0 × 10^−4^); and it was 8.55 × 10^−8^ for candle combustion, suggesting that the carcinogenic risk can be neglected. The carcinogenic risk of As and Ni from cigarette combustion was similar to 1.57 × 10^−5^ and 7.73 × 10^−5^, respectively, while that of Cr was higher than 4.19 × 10^−4^ (61). Consistent with the previous study (62), BaP contributed mainly to the carcinogenic risk of c-PAHs, with values of 1.21 × 10^−5^ and 1.06 × 10^−4^ for mosquito coil and cigarette combustion, respectively, while it could not be detected in candle combustion. Meanwhile, the values of the other three PAHs (BaP, Chr, and BbF) were all lower than 1.0 × 10^−4^. Both heavy metals and c-PAHs from indoor combustion emissions might increase the potential carcinogenic risk, especially Cr and BaP, both of which should cause more public attention.

## 4 Conclusion

This study experimentally illustrates the emission characteristics and health risks of the PM constituents emitted from indoor combustion sources. The concentration and composition of the PM emitted from three combustions revealed the following trend: cigarette > mosquito coil > candle. However, no Flo substances were detected in candle burning. The results demonstrated that mosquito coil, cigarette, and candle were important emission sources of indoor PM, and the burning of cigarette and mosquito coil may generate higher emissions than candle burning. The size distribution of both DOM and PAHs was concentrated in the range of 0.18–0.32 μm across 10 size distributions and mostly showed that the concentrations in the fine particles were much higher than those in the coarse size, suggesting that these substances were more likely to accumulate in fine fractions. Since fine particles are easier to reach human body, this size distribution feature might cause the PM indoors to have a great impact on human health. This highlights that the chemical composition of PM is not the only factor affecting health but properties including its size must also be factored into consideration. The results of the characteristic ratios of PAHs in PM reveal that the mosquito coil and cigarette indoor combustion types belong to biomass combustion and the candle combustion points to a petroleum type. Furthermore, the characteristic ratio of Flu/(Flu + Pyr) at 0.2 can serve as a precise indicator to distinguish between mosquito coil and cigarette origins.

The carcinogenic risk assessment results showed that the comprehensive carcinogenic risk of heavy metals was 6.14 × 10^−1^, 5.18 × 10^−1^, and 5.69 × 10^−1^ corresponding to mosquito coil, cigarette, and candle combustion, wherein all exceeded the tolerance level. The comprehensive carcinogenic risk of PAHs from three combustions was in the range of 8.55 × 10^−8^ to 1.46 × 10^−5^ and was also above the safety level of humans except candle combustion. This indicates that long-term exposure to this environment is likely to lead to an increase in potential carcinogenic risks, which reminds the public to pay more attention to non-open-flame combustion sources than open flame combustion indoors.

## Data Availability

The original contributions presented in the study are included in the article/Supplementary material, further inquiries can be directed to the corresponding author.
