# Supplementary material for: Characteristics of airborne particles emitted from typical indoor combustion sources
Source: Front Public Health. 2025 Feb 19;13:1540166. doi: 10.3389/fpubh.2025.1540166 (PMC11880257; doi:10.3389/fpubh.2025.1540166)
Supplement: Supplementary file 1 [file Supplementary_file_1.pdf]

Characteristics of airborne particles emitted from typical indoor combustion sources

Chen Geng [a,1] , Xinyuan Wu [a,1], Tao Wang [a], Hongbo Fu [a,b,c]

[a] Shanghai Key Laboratory of Atmospheric Particle Pollution and Prevention, Department of Environmental Science & Engineering, Institute of Atmospheric Sciences, Fudan University, Shanghai 200433, PR China

[b] Collaborative Innovation Centre of Atmospheric Environment and Equipment Technology (CICAEET), Nanjing University of Information Science and Technology, Nanjing 210044, PR China

[c] Institute of Eco–Chongming (SIEC), 20 Cuiniao Road, Chenjia Town, Chongming District, Shanghai, 202162, PR China

[*]Correspondence: fuhb@fudan.edu.cn

[1]Both authors contributed equally as first author.

Materials and methods

Initially, this experiment entailed a continuous 24-hour monitoring of indoor background air in a setting devoid of combustion, during which period blank samples were meticulously collected to deduct background values. Subsequently, indoor burning experiments were carried out with mosquito coils, cigarettes, and candles, respectively, while ensuring that all doors and windows remained closed during the experiment. Each group is set up with three parallel experiments, allowing them to burn freely and timing, and stopping burning after 1 hour. Three types of PM samples emitted from combustion sources were collected for composition analysis. In this study, well-known and widely used mosquito coils and cigarette brands were selected, namely Lanju and Huanghe Tower, and the candles used in the combustion experiment were selected as lighting candles made of refined paraffin wax and pure cotton wick material. The consumption of mosquito coils, cigarettes, and candles burned within 1 hour is about half to one-third of a plate of mosquito coils, 6 cigarettes, and 1-2 candles, respectively.

Before initiating the experiment, the experimental room underwent a thorough and meticulous cleaning. During the combustion experiments, identical types of combustion sources were scheduled to burn within a single day. Following the completion of each set of experiments, an overnight ventilation period was implemented to thoroughly purge any residual effects, thereby eliminate any potential interference among the three combustion sources. In experiments focusing on the same type of combustion source, a ventilation period of one hour at maximum flow rate is ensured after the combustion has ceased, ensuring that the indoor air quality was restored and prepared for the next experiment. Except for ventilation experiments, the doors and windows of the office were kept firmly closed throughout the entire testing period to minimize interference from external factors.

Tab. S1. Carcinogenic risk parameters of the metals and PAHs.

	IUR[1] $((\mu g \cdot m^{-3})^{-1})$	ET[2] $(h \cdot d^{-1})$	EF[3] $(d \cdot year^{-1})$	ED (year)[3]	AT (d)[3]
Cr	8.4×10^{-2}				
Cd	1.8×10^{-3}				
Ni	2.6×10^{-4}				
Pb	8.0×10^{-5}				
As	4.3×10^{-3}	1.38	255	53	25550
Benz[a]anthracene	1.1×10^{-4}				
Chrysene	1.1×10^{-5}				
Benzo[b]fluoranthene	1.1×10^{-4}				
Benzo[a]pyrene	1.1×10^{-3}				

Tab. S2. Sum emission factor of 18 kinds of metals emitted from three combustion processes (μg·g^{-1})

	Ns	Mg	K	Ca	Hg	Mo	Ba	Cr	Pb	As	Mn	Ni	V	Co	Ag	Cd	Sb	U
Mosquito coil	4685.40	1401.55	630.22	1899.81	1819.05	9.48	9.65	5.39	3.20	1.53	2.33	2.50	0.26	0.06	0.41	0.05	0.29	0.16
cigarette	4899.32	3799.57	723.79	2377.38	1403.03	10.06	21.49	4.66	3.29	1.55	3.54	2.86	0.58	0.57	0.17	0.15	0.45	0.20
candle	2066.50	664.43	303.06	1403.03	661.83	6.19	6.53	2.84	1.06	0.95	1.16	1.29	0.38	0.05	0.47	0.03	0.19	0.10

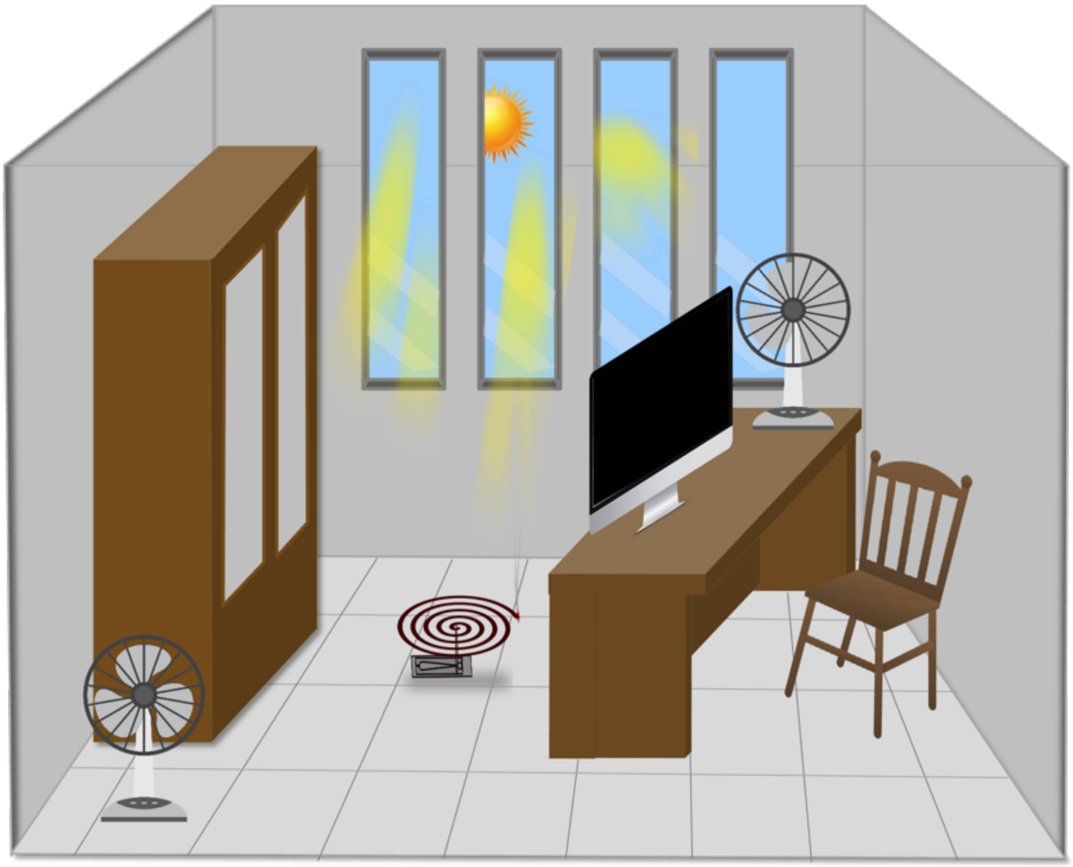

Fig. S1. Sampling diagram for indoor combustion experiment.

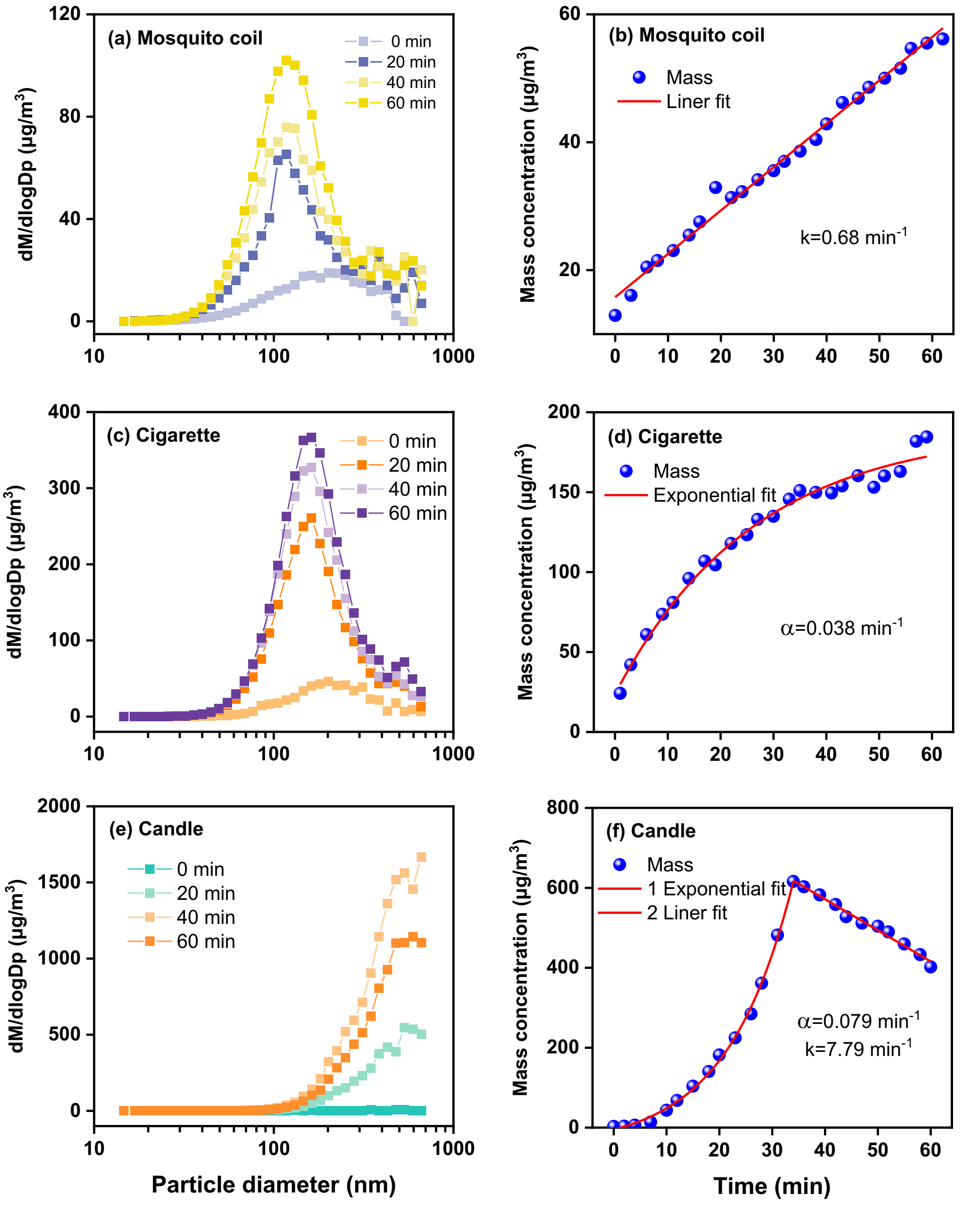

Fig. S2. Size-resolved PM mass concentration during (a) mosquito coil, (c) cigarette, (e) candle combustion and fitted lines for (b) mosquito coil, (d) cigarette, (f) candle combustion as a function of measurement time.

References

[1] USEPA. Regional Screening Level (RSL) Resident Air Supporting Table [Z]. 2011a.

[2] Zhou J, Han B, Bai Z, et al. Particle Exposure Assessment for Community Elderly (PEACE) in Tianjin, China: Mass concentration relationships [J]. Atmospheric Environment, 2012, 49: 77-84.

[3] Chen J W, Wang S L, Hsieh D P H, et al. Carcinogenic potencies of polycyclic aromatic hydrocarbons for back-door neighbors of restaurants with cooking emissions [J]. Science of the Total Environment, 2012, 417: 68-75.
